# Behavioral biology of *Toxoplasma gondii* infection

**DOI:** 10.1186/s13071-020-04528-x

**Published:** 2021-01-25

**Authors:** Wen Han Tong, Chris Pavey, Ryan O’Handley, Ajai Vyas

**Affiliations:** 1 School of Biological Sciences, Nanyang Technological University (SBS-NTU), 60 Nanyang Drive, Singapore, 637551 Singapore; 2 Commonwealth Scientific and Industrial Research Organisation (CSIRO) Land and Water, Darwin, Australia; 3 School of Animal and Veterinary Sciences, University of Adelaide, Roseworthy Campus, Roseworthy, Australia

**Keywords:** Apicomplexa, Behavioral manipulation, Complex life cycle, Extended phenotypes, Parasitic manipulation, Protozoa

## Abstract

*Toxoplasma gondii* is a protozoan parasite with a complex life cycle and a cosmopolitan host range. The asexual part of its life cycle can be perpetually sustained in a variety of intermediate hosts through a combination of carnivory and vertical transmission. However, *T. gondii* produces gametes only in felids after the predation of infected intermediate hosts. The parasite changes the behavior of its intermediate hosts by reducing their innate fear to cat odors and thereby plausibly increasing the probability that the definitive host will devour the infected host. Here, we provide a short description of such parasitic behavioral manipulation in laboratory rodents infected with *T. gondii*, along with a bird’s eye view of underpinning biological changes in the host. We also summarize critical gaps and opportunities for future research in this exciting research area with broad implications in the transdisciplinary study of host–parasite relationships.

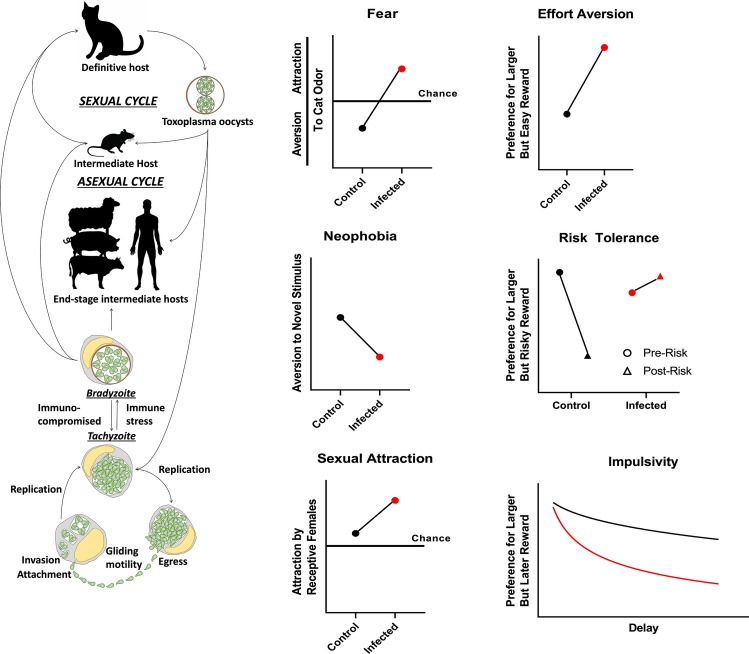

## What is *Toxoplasma gondii*?

*Toxoplasma gondii* is an enteric coccidian parasite of the cat. As an apicomplexan protist, *T. gondii* is an intracellular parasite that completes its life cycle within the small intestine of the cat, which is the definitive host, where the parasite undergoes sexual reproduction to produce oocysts. These unsporulated oocysts, which measure 11–13 μm in diameter, are excreted in the infected cat’s feces. Following development in the environment, termed sporulation, the sporulated oocysts that are ingested by an intermediate host release sporozoites, and these infect the cells of the intestine and lymph nodes where they transform into a rapidly dividing asexual stage termed a tachyzoite. The tachyzoites disseminate to other parts of the body, and their rapid multiplication and associated destruction of host cells result in acute toxoplasmosis, which is characterized by mild to severe clinical signs depending on the organ system affected and the immune status of the host. Following this acute stage, the formation of tissue cysts containing a slow reproducing bradyzoite stage occurs. These tissue cysts, which typically form in the brain, liver and muscle, can persist for the life of the intermediate host. Upon ingestion by the cat, bradyzoites are released from tissue cysts and undergo asexual reproduction within the epithelium of the cat small intestine, followed by sexual reproduction and the production of oocysts. However, if other intermediate hosts ingest the tissue cysts, the acute stage of the life cycle, characterized by tachyzoite replication and dissemination, is initiated. If infection occurs during pregnancy, tachyzoites can be transmitted vertically to the fetus. They may be associated with severe clinical outcomes depending on the timing of the infection and the host species involved.

*Toxoplasma gondii* is considered to be one of the most “successful” parasites since virtually any warm-blooded animal can serve as a paratenic host. In humans, exposure is frequent, with estimated exposure rates of between 30 and 35% in the general population [1]. However, exposure rates are significantly influenced by dietary habits and behavior and can range from 10 to 80% in specific populations [2, 3].

## Effects on host behavior

If a laboratory-reared rat is placed in a box with a few drops of cat urine in one corner it will avoid the corner with the cat urine. The rat will also secrete stress hormones, increase vigilance, and be less likely to feel pain. Rats have an innate fear of cues of cats being nearby. Now, if a rat similar in all aspects with the first rat with the exception it has been previously infected with *T. gondii* is placed in the same box, this infected rat does not avoid the corner with the urine [4, 5]. Some infected rats are even attracted to the odor. In other words, *T. gondii* removes the innate fear of rats and converts it, in at least some individuals, into attraction, a feat which decades of laboratory-rearing sans-predator has not yet achieved. Why does it happen? The most popular explanation is that a fearless rat is more likely to be eaten by a cat, which is beneficial for *T. gondii* because it forms gametes in the cat, the definitive host, and is then excreted in the feces in the form of environmentally stable oocysts. Thus, *T. gondii* manipulates the behavior of its host to increase its transmission. The rodent–*T. gondii* model is, in fact, one of the most studied models of parasitic behavioral manipulation [6–12].

However, there is a wide range of views on just how specific the behavioral effects of *T. gondii* infection are. These can be broadly classified into three views. First, a set of studies suggest that *T. gondii* effects are rather specific to the avoidance of cat odors, with odors of non-predators, or predators not important for the parasite’s life cycle being less influenced by the manipulative effects of the parasite [5, 13]. A second set of studies reports that the effects are syndromic, in that infection causes a loss of a suite of host defensive behaviors. Thus, trappability increases even if human-made traps are not similar to cats. Infected rats become more exploratory, more open to taking risks and become altogether more impulsive [14–21]. Finally, a third set of studies suggests that *T. gondii* causes something akin to a behavioral fever that includes a broad range of behavioral changes that are ill-suited for the host, and that the combination of these changes might increase predation by cats [22–29]. Why is there such a diversity of notions? A large part of this diversity comes from the specific strains of *T. gondii* and rodent species being used the study. The majority of genetic diversity found in *T. gondii * can be found in three clonal lineages [30]. Of these, infection of rats with the less virulent type II strains typically leads to smaller magnitude of sickness behaviors during the acute phase of the infection. These infections, in turn, cause more specific behavioral effects during the chronic period. Accordingly, *T. gondii* infection of outbred rats or infection with low-virulence parasites produce remarkably specific behavioral effects with minimal sickness behavior [4, 5, 31]. On the other hand, infection of inbred mice with virulent type I parasite strains causes much acute sickness and less specific behavioral effects [22, 28, 32, 33]. Thus, the behavioral outcome of *T. gondii* infection systematically varies with the host–parasite strain combinations.

## Why study the behavioral biology of *T. gondii*?

Biologists have been long interested in the relationship between parasitism and host behavior. Continual coevolution with parasites, for example, has been used to explain the persistence of heritable sexual advertisements in males [34]. Similarly, the concept of extended phenotypes often uses behavioral manipulation of the host by parasites as a rhetoric device to argue that natural selection acts on genetic information and not on individuals [35]. Several parasites change the behavior of their hosts, presumably to increase the transmission efficiency. Thus, genes of parasites can cause a phenotype outside of the physical confines of the individual body of the parasite; for that reason, selection operates on the genetic information of the parasite rather than on its physical individuality. *T. gondii* infection of laboratory rodents provides an experimentally tractable system in which testable hypotheses about extended phenotypes can be examined.

Also, behavioral neurobiologists can use *T. gondii* as a perturbation model to understand innate fear in much the same way as a geneticist might use naturally occurring allelic mutation. This is a critical pursuit because the biology of fear relates to the biology of psychiatric diseases, such as posttraumatic stress disorder. Studies on *T. gondii* behavioral biology have led to several exciting discoveries in neuroendocrinology, including the organization of brain–hormone communication (e.g. [36]).

Medical research on *T. gondii* was once restricted to immune-compromised patients. However, the discovery of behavioral changes in *T. gondii*-infected humans has slowly revised that focus. Concordance studies show that *T. gondii* increases the odds of developing psychiatric disorders such as schizophrenia, bipolar disorder and obsessive-compulsive disorder [37, 38]. These retrospective estimates have been confirmed by prospective studies in the case of schizophrenia [39, 40] (but see lack of evidence in a birth cohort study [41]). Multiple case–control studies also show that latent *T. gondii* infection is associated with systematic changes in human personality [42, 43], ranging from risk-taking to entrepreneurship [44, 45].

Manipulative parasites represent an exciting gap in the study of ecological systems [46]. For example, prey–predator interactions can often lead to trophic cascades, indirect effects that percolate to resources consumed by the prey. Manipulative parasites like *T. gondii* are likely to affect such cascades due to the creation of heterogeneity in the probability of consumption of infected versus non-infected prey. Similarly, the specificity of *T. gondii* behavior effects, specifically definitive* versus* dead-end host, might influence prey switching by generalist predators like raptors and owls [47].

*Toxoplasma gondii* and its attendant effects of host behavior thus provide a unique transdisciplinary intersection between diverse fundamental and applied biological disciplines.

## Three advances in *T. gondii* behavioral biology

### *Toxoplasma gondii* infection alters host hormones

*Toxoplasma gondii* exhibits a decided tropism for the brain and eyes. These organs are immune privileged in that they afford limited access to immune cells. Interestingly, the brain and eyes are not the only immune-privileged sites in the mammalian body. Male testes segregate developing sperms behind a tight barrier that largely excludes the entry of immune cells. Very few pathogens succeed in crossing the blood–testes barrier; yet *T. gondii* does cross this barrier and invades testes in rats [48]. Accordingly, *T. gondii* is found in the ejaculates of rats and several other species [48–52] (but not in mice [28]). Moreover, *T. gondii* increases the synthesis of testosterone [53], a testicular hormone that reduces fear and anxiety [54]. The extra testosterone is crucial for *T. gondii* effects, primarily through its interaction with the brain. Castration prevents behavioral change, and *T. gondii*-like effects can be recapitulated by the infusion of testosterone within the host brain [53]. This raises an obvious but unanswered question: how does *T. gondii* change the behavior of testes-free females [55]?

### *Toxoplasma gondii* infection alters neurotransmission within the host brain

Chronic *T. gondii* infection causes specific changes in chemical messengers used by inter-neuronal connections within the brain. The *T. gondii* genome contains two loci with high homology for mammalian genes coding for a rate-limiting enzyme in dopamine synthesis, namely amino acid hydroxylase [56]. This homology suggests that *T. gondii*, once resident within the brain, can increase the availability of dopamine [57, 58], a neurotransmitter critical for the processing of motivation and pleasure. Interestingly, drugs that interfere with dopamine metabolism also rescue the effects of *T. gondii* on behavior [59]. Nonetheless, genetic ablation of at least one of these genes does not rescue *T. gondii*-induced behavioral manipulation [60, 61]. *Toxoplasma gondii* infection also increases the synthesis of arginine vasopressin in a specific brain region called the medial amygdala [62]. These neurons are involved in the perception of sexual pheromone and connected to brain regions involved in motivation [36]. One proposal is, therefore, that the extra dopamine or arginine vasopressin or both cause animals to become impulsive and reckless, leading to a reduction in fear.

### *Toxoplasma gondii* effects could be mediated through neuroinflammation

The host immune system is critical in limiting the acute phase of *T. gondii* infection and then maintaining encystment during repeated cycles of recrudescence. Results from several studies suggest that inflammation also plays an essential role in mediating behavioral effects. For example, the number of cysts in the mouse brain and its attendant immune response correlates with the magnitude of behavioral change [22]. Similarly, an anti-inflammatory drug, guanabenz, reduces the behavioral effects of the infection [63]. The authors of these studies argue that the behavioral manipulation is not as specific to cat odors as initially suggested, but is instead part of a multi-faceted alteration caused by the continual immune challenge. Yet, *T. gondii* strains that do not cause sustained encystment in the mouse brain still able to manipulate behavior [31]. Moreover, it is possible to rescue behavioral effects by blocking molecular changes within restricted brain regions, a treatment unlikely to affect cyst density [62]. Specific* versus* aspecific effects of *T. gondii* likely reflect molecular traits specific to the host species.

## Three opportune research areas

### Consequences of the infection on predation rates

The central theme of the behavioral manipulation hypothesis is that parasites can change host behavior and that this change has a current adaptive value for the parasite. While there are several examples of host behavioral change [64], the adaptive value for the parasites remains unquantified in the vast majority of these cases. Apropos, it is unknown if *T. gondii* increases predation rates of the infected rodents by cats, and if the possible facilitation generalizes to non-cat predators, such as birds of prey. *Toxoplasma gondii* can perpetually maintain the asexual phase of its life cycle without cats [65], while gametogenesis requires cats, although genetic heterogeneity of the parasite and benefits reaped by recombination remain doubtful. Nonetheless, cats provide an opportunity to infect herbivores as well as the host for the generation of copious amounts of environmentally resistant oocysts. In terms of behavioral facilitation of predation, therefore, it is critical to understand both the behavioral manipulation hypothesis and the relative strengths of the transmission modes. In this context, it is crucial to ascertain whether *T. gondii*-infected rodents are consumed at a higher rate by cats than non-infected individuals.

### Consequences of the infection for life-history plasticity

An increase in parasite-mediated predation can have important implications for host life-history traits, including the relative balance between current and residual reproduction. Animals respond to increased risk of death by creating myopic life histories that favor earlier reproduction even if this is a trade-off with survival [66]. For example, Tasmanian devils reproduce earlier in response to facial tumor disease as the probability of being alive during second or third episodes of reproduction becomes constrained [67]. If *T. gondii* increases predation rates, then it is likely that the host will respond with abbreviated life histories. Such adjustments will alter ecological interactions at the community level and reconfigure the flow of energy through trophic levels. Thus, the critical unanswered questions include: does *T. gondii* alter life-history traits; does such change reflect phenotypic plasticity or genetic heterogeneity within hosts; and do these non-consumptive changes in the host percolate through ecological communities? Focusing on answering such questions is also an opportunity to understand the theoretical relationships between the effects of parasitism and non-parasitic clinical conditions [68].

### Making sense of diverse host–parasite combinations

Experiments show a large variability in behavioral effects of *T. gondii*, especially between studies using rats and mice. It remains unknown if this variability reflects a genetic divergence in species or represents a result of recent inbreeding in laboratory strains. Behavioral studies using other hosts are rare to find. This gap is a significant research opportunity to build phylogenetic comparisons of behavioral responses to the infection. There is a need to demonstrate convergence and divergence across phylogenetic branches in order to demonstrate the historical processes of selection on the parasite. Such systematic comparisons are also crucial to improving our understanding of the implications of parasitism on the evolution of host behavior, for example, to answer if manipulative and non-manipulative parasites provide different pressures on host sexual selection. The differential effects of *T. gondii* strains on host behavior are another provocative area of research in which the use of genetic crosses of the parasite may help to map relevant loci in the parasite genome.

## Conclusions

More than five decades ago, Niko Tinbergen articulated four crucial vantage points to understanding behavior [69]: mechanisms, development, current adaptive value and evolutionary origin. This framework has often been used as a scaffold on which to organize scientific knowledge in behavior and associated disciplines [70]. The same structure can serve well to organize current advances and areas ripe for future research on *T. gondii* behavioral biology. Starting from a careful demonstration of parasitic host behavioral change, the model has slowly dovetailed into detailed experiments about the mechanisms, the first player in Tinbergen’s quartet. We have not yet identified the *T. gondii* genes or effector proteins responsible for parasitic behavioral change. Nevertheless, considerable progress has been made in the domain of downstream neuroendocrine changes within the host. The other three players in Tinbergen’s quartet, namely developmental plasticity, adaptive value and evolutionary origins, remain unexplored areas ripe for future investigation. Parasites are robust drivers of host behavior, with cardinal influences in anti-predator defense and sexually selected traits. Hence, the elucidation of *T. gondii* behavioral biology has broad implications in the fields of neurobiology, behavioral ecology and evolutionary biology. This research enterprise is likely to see much scientific attention in the future.

A downloadable poster describing the behavioral biology of *T. gondii * is available in Additional file 1: Poster S1.

## Supplementary Information

**Additional file 1: Poster S1.** Poster describing the behavioral biology of *T. gondii *.

## Data Availability

Not applicable.
