# Supplementary material for: Behavioral biology of Toxoplasma gondii infection
Source: Parasit Vectors. 2021 Jan 25;14:77. doi: 10.1186/s13071-020-04528-x (PMC7831251; doi:10.1186/s13071-020-04528-x)
Supplement: Supplementary file 1 — Additional file 1: Poster S1. Poster describing the behavioral biology of T. gondii . [file 13071_2020_4528_MOESM1_ESM.pptx]

## Slide 1
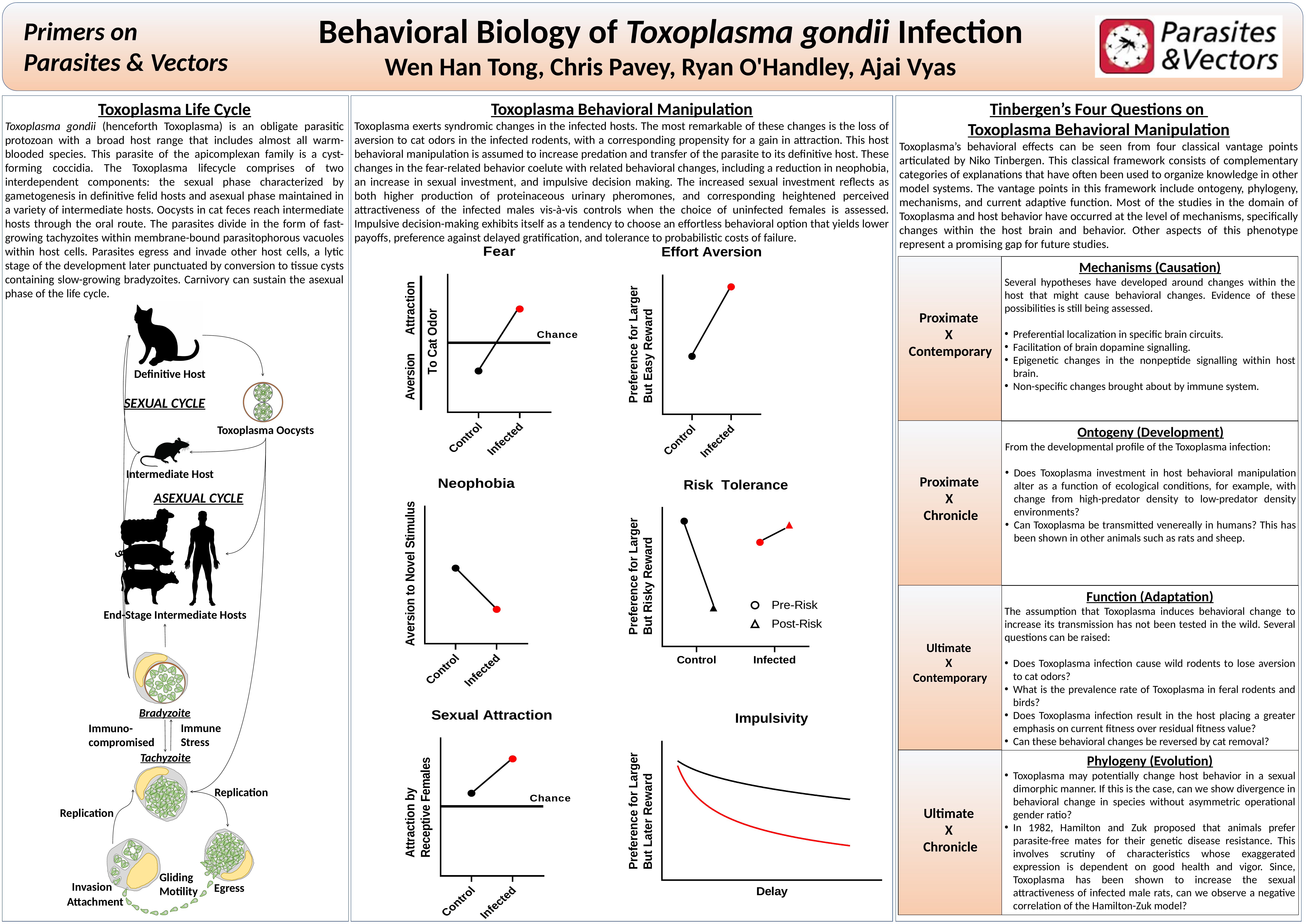

Behavioral Biology of Toxoplasma gondii Infection
Wen Han Tong, Chris Pavey, Ryan O'Handley, Ajai Vyas
Primers on
Parasites & Vectors
Toxoplasma Behavioral Manipulation
Toxoplasma exerts syndromic changes in the infected hosts. The most remarkable of these changes is the loss of aversion to cat odors in the infected rodents, with a corresponding propensity for a gain in attraction. This host behavioral manipulation is assumed to increase predation and transfer of the parasite to its definitive host. These changes in the fear-related behavior coelute with related behavioral changes, including a reduction in neophobia, an increase in sexual investment, and impulsive decision making. The increased sexual investment reflects as both higher production of proteinaceous urinary pheromones, and corresponding heightened perceived attractiveness of the infected males vis-à-vis controls when the choice of uninfected females is assessed. Impulsive decision-making exhibits itself as a tendency to choose an effortless behavioral option that yields lower payoffs, preference against delayed gratification, and tolerance to probabilistic costs of failure.
Tinbergen’s Four Questions on
Toxoplasma Behavioral Manipulation
Toxoplasma’s behavioral effects can be seen from four classical vantage points articulated by Niko Tinbergen. This classical framework consists of complementary categories of explanations that have often been used to organize knowledge in other model systems. The vantage points in this framework include ontogeny, phylogeny, mechanisms, and current adaptive function. Most of the studies in the domain of Toxoplasma and host behavior have occurred at the level of mechanisms, specifically changes within the host brain and behavior. Other aspects of this phenotype represent a promising gap for future studies.
Toxoplasma Life Cycle
Toxoplasma gondii (henceforth Toxoplasma) is an obligate parasitic protozoan with a broad host range that includes almost all warm-blooded species. This parasite of the apicomplexan family is a cyst-forming coccidia. The Toxoplasma lifecycle comprises of two interdependent components: the sexual phase characterized by gametogenesis in definitive felid hosts and asexual phase maintained in a variety of intermediate hosts. Oocysts in cat feces reach intermediate hosts through the oral route. The parasites divide in the form of fast-growing tachyzoites within membrane-bound parasitophorous vacuoles within host cells. Parasites egress and invade other host cells, a lytic stage of the development later punctuated by conversion to tissue cysts containing slow-growing bradyzoites. Carnivory can sustain the asexual phase of the life cycle.
Mechanisms (Causation)
Several hypotheses have developed around changes within the host that might cause behavioral changes. Evidence of these possibilities is still being assessed.
Preferential localization in specific brain circuits.
Facilitation of brain dopamine signalling.
Epigenetic changes in the nonpeptide signalling within host brain.
Non-specific changes brought about by immune system.
Proximate
X
Contemporary
Ontogeny (Development)
From the developmental profile of the Toxoplasma infection:
Does Toxoplasma investment in host behavioral manipulation alter as a function of ecological conditions, for example, with change from high-predator density to low-predator density environments?
Can Toxoplasma be transmitted venereally in humans? This has been shown in other animals such as rats and sheep.
Proximate
X
Chronicle
Function (Adaptation)
The assumption that Toxoplasma induces behavioral change to increase its transmission has not been tested in the wild. Several questions can be raised:
Does Toxoplasma infection cause wild rodents to lose aversion to cat odors?
What is the prevalence rate of Toxoplasma in feral rodents and birds?
Does Toxoplasma infection result in the host placing a greater emphasis on current fitness over residual fitness value?
Can these behavioral changes be reversed by cat removal?
Ultimate
X
Contemporary
Phylogeny (Evolution)
Toxoplasma may potentially change host behavior in a sexual dimorphic manner. If this is the case, can we show divergence in behavioral change in species without asymmetric operational gender ratio?
In 1982, Hamilton and Zuk proposed that animals prefer parasite-free mates for their genetic disease resistance. This involves scrutiny of characteristics whose exaggerated expression is dependent on good health and vigor. Since, Toxoplasma has been shown to increase the sexual attractiveness of infected male rats, can we observe a negative correlation of the Hamilton-Zuk model?
Ultimate
X
Chronicle
Definitive Host
Toxoplasma Oocysts
Intermediate Host
End-Stage Intermediate Hosts
Bradyzoite
Immune
Stress
Immuno-
compromised
Tachyzoite
Replication
Replication
Gliding
Motility
Invasion
Egress
Attachment
SEXUAL CYCLE
ASEXUAL CYCLE
